# IS*Aba1*-mediated *bla*_ADC_ disruption restores third-generation cephalosporin susceptibility in a clinical *Acinetobacter baumannii* isolate

**DOI:** 10.1128/aac.00707-25

**Published:** 2025-11-18

**Authors:** Carolina S. Nodari, Qinqin Bai, Rodrigo Cayô, Ana Paula Streling, Felipe Alberto-Lei, Julia Wille, Antonio C. C. Pignatari, Harald Seifert, Ana Cristina Gales, Paul G. Higgins

**Affiliations:** 1 Laboratório Alerta, Division of Infectious Diseases, Department of Internal Medicine, Universidade Federal de São Paulo (UNIFESP) 28105, São Paulo, Brazil; 2 Institute for Medical Microbiology, Immunology and Hygiene, Faculty of Medicine and University Hospital Cologne, University of Cologne 14309, Cologne, Germany; 3 Department of Public Health Laboratory Sciences, School of Public Health, Hengyang Medical School, University of South China 34706, Hengyang, China; 4 Universidade Federal de São Paulo (UNIFESP), Laboratório de Resistência Antimicrobiana Ambiental (LEARN), Setor de Biologia Molecular, Microbiologia e Imunologia, Departamento de Ciências Biológicas (DCB), Instituto de Ciências Ambientais, Químicas e Farmacêuticas (ICAQF) 734878, Diadema, Brazil; 5 Antimicrobial Resistance Institute of São Paulo (ARIES), São Paulo, Brazil; 6 German Center for Infection Research (DZIF), partner site Bonn-Cologne, Cologne, Germany; 7 Laboratório Especial de Microbiologia Clínica (LEMC), Division of Infectious Diseases, Department of Internal Medicine, Universidade Federal de São Paulo (UNIFESP) 28105, São Paulo, Brazil; 8 Institute of Translational Research, Cologne Excellence Cluster on Cellular Stress Responses in Aging-Associated Diseases (CECAD), Faculty of Medicine and University Hospital Cologne, University of Cologne 14309, Cologne, Germany; Universita degli studi di roma La Sapienza, Rome, Italy

**Keywords:** insertion sequences, *Acinetobacter*-derived cephalosporinase, carbapenem resistance, colistin resistance

## Abstract

This study describes the effects of the insertion of IS*Aba1* in the intrinsic *bla*_ADC_ gene of an *Acinetobacter baumannii* isolate showing co-resistance to carbapenems and colistin. Isolate 52971 showed low ceftazidime MIC values due to the disruption of *bla*_ADC-195_ by this insertion sequence. The introduction of an intact *bla*_ADC_ in isolate 52971 increased ceftazidime MIC. The potential of insertion sequences to inactivate chromosome-encoded resistance determinants should be explored.

## INTRODUCTION

IS*Aba1* is an insertion sequence (IS) often present in multiple copies in the genome of *Acinetobacter baumannii* (1). It can promote antimicrobial resistance by either providing a strong promoter when inserted upstream of a gene in its opposing transcriptional orientation (2, 3) or by disrupting protein-encoding genes such as those for the outer membrane (4) or gene regulators (5). The presence of IS*Aba1* upstream of *bla*_ADC_-like genes, the intrinsic AmpC encoding gene in *Acinetobacter* spp., is strongly associated with high cephalosporin MICs in *A. baumannii* (6). However, the role of this IS in restoring cephalosporin susceptibility has not, to the best of our knowledge, been previously reported. The present study describes the disruption of *bla*_ADC-195_ by IS*Aba1*, in a carbapenem-resistant *A. baumannii* clinical isolate, resulting in ceftazidime susceptibility despite the presence of an IS*Aba1*-associated strong promoter upstream of this beta-lactamase encoding gene.

*A. baumannii* isolate 52971 was recovered from a soft-tissue specimen in May 2012 in São Paulo, Brazil. Antimicrobial susceptibility testing (AST) was performed by broth microdilution using cation-adjusted Mueller-Hinton broth following ISO 20776 guidelines. MIC values for ceftazidime, imipenem, meropenem, ciprofloxacin, gentamicin, amikacin, minocycline, tigecycline, colistin, and polymyxin B (Sigma-Aldrich, St. Louis, USA) were interpreted using Brazilian Committee on Antimicrobial Susceptibility Testing (BrCAST/EUCAST) clinical breakpoints version 12.0 (2022), when available (http://brcast.org.br/documentos/). AST revealed that isolate 52971 presented high MICs to carbapenems (MIC, 128 mg/L), ciprofloxacin (MIC, >64 mg/L), tigecycline (MIC, 16 mg/L), gentamicin (MIC, 32 mg/L), and polymyxins (polymyxin B and colistin MICs, 32 and >128 mg/L, respectively), but remained susceptible to amikacin (MIC, 8 mg/L), and showed low MICs for minocycline (MIC, 2 mg/L) and ceftazidime (MIC, 1 mg/L) (Table S1).

Whole-genome sequencing (WGS) of *A. baumannii* 52971 was performed with Illumina MiSeq (Illumina Inc., San Diego, USA), reads were assembled with SPAdes (7), and resistome and IS elements were identified with a combination of online tools and manual curation, as previously described (8). Isolate 52971 presented a genome of 3,948,062 bp and clustered with international clone (IC) 1 (clonal complex^Pas/Oxf^ 1/109) (Table S2). Resistome analysis demonstrated a strong correlation with the phenotype presented by the isolate, which harbored acquired resistance determinants to carbapenems (IS*Aba1+bla*_OXA-23_), aminoglycosides [*aadA1*, *aac(3)-Ia*, and *aph(3’)-Ic*], and sulfonamides (*sul1*). Additionally, amino acid substitutions associated with resistance to fluoroquinolone [GyrA (S83L, E87G) and ParC (S84L)], tigecycline [AdeS (L100N, T156M)], and colistin [PmrC (L124S)] were observed (Table S1).

**Fig 1:**
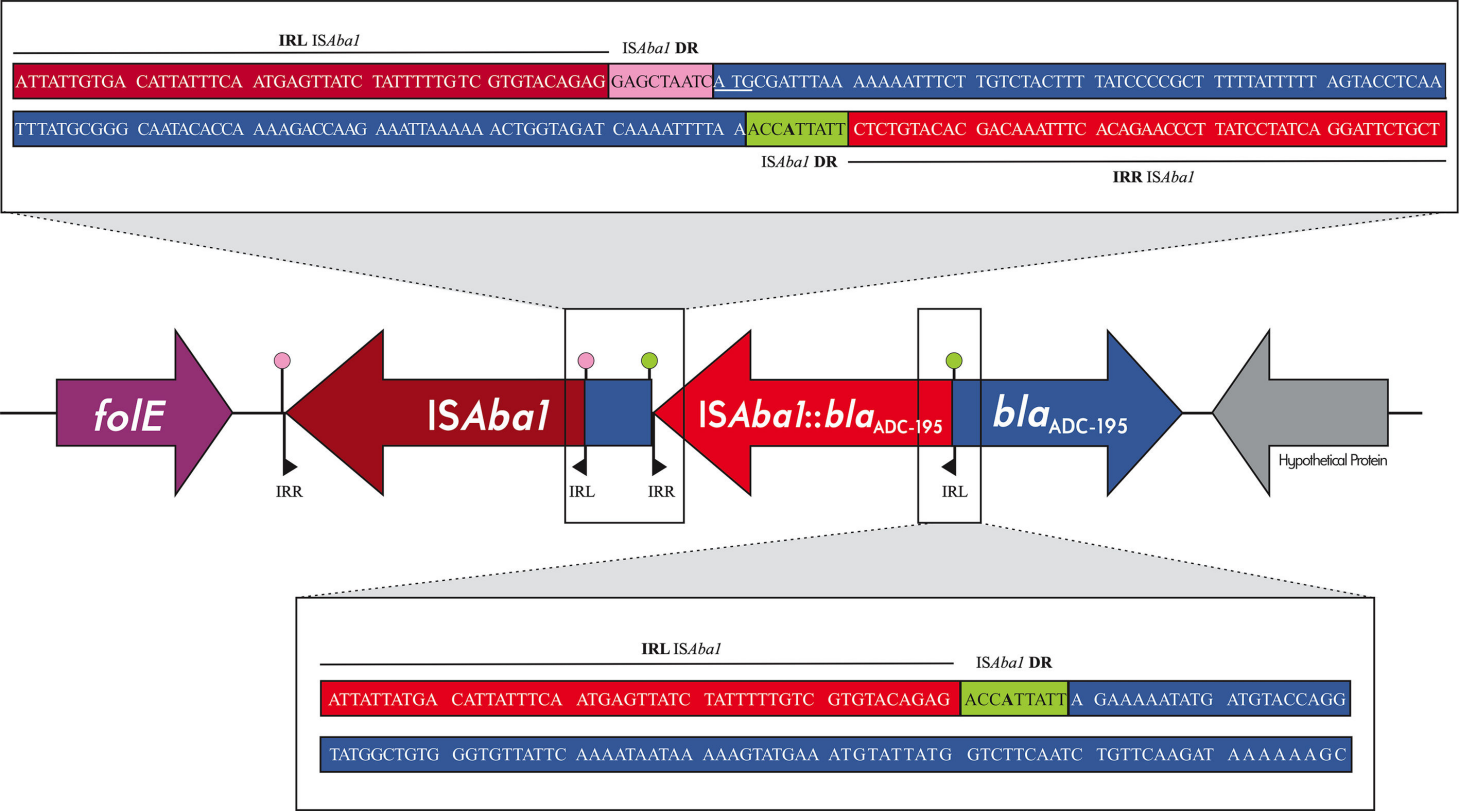
Genetic context of *bla*_ADC-195_ (in blue) in *A. baumannii* isolate 52971. IS*Aba1* (in red) insertion sites and direct repeats (DRs, in pink and green) are highlighted in the boxes. The G126A substitution in the target site is indicated in bold. Figure is not to scale.

The low ceftazidime MIC of the isolate suggested that some alterations of the chromosomal *bla*_ADC_ gene could be present; either it was not expressed or had lost its activity against third-generation cephalosporins. Sequence analysis revealed that the *bla*_ADC_-like gene was disrupted by the insertion of IS*Aba1* in an inverted transcriptional orientation after nucleotide T131 (Fig. 1). The insertion of IS*Aba1* also led to a 9 bp duplication of the target insertion site (ACCATTATT). Such disruption created an abnormal and shorter (only 80 out of the expected 383 amino acids) translated protein lacking the amino acid motifs associated with ADC enzymatic activity (Fig. S1).

*In silico* removal of IS*Aba1* allowed us to identify the ADC variant as ADC-195, which presented all the motifs associated with beta-lactamase activity and no amino acid substitutions in the H-2 and H-10 helices, which were previously found to be associated with extended-spectrum activity of AmpC enzymes (Fig. S1) (9). ADC-195 is closely related (>99% protein identity) to ADC-183 and ADC-5, differing from them by two and three amino acid substitutions, respectively (Fig. S1). It should be noted that the amino acid substitution A236V is located within the Ω-loop of the protein, which plays an essential role in beta-lactamase activity and was previously found associated with increased ceftazidime hydrolysis (9). However, even though this was not confirmed *in vitro*, none of the amino changes observed when comparing ADC-195 to ADC-183 should affect its hydrolysis profile since the substitutions (A236V and S341T) did not alter the charge (both amino acids are neutral), their (hydrophobic) character, or the size of the lateral chain. A copy of IS*Aba1* was also found upstream of *bla*_ADC-195_ in an inverted transcriptional orientation (Fig. 1), potentially providing a strong promoter, as previously described, which would contribute to its overexpression and lead to higher cephalosporin MICs (6).

Therefore, it was hypothesized that cloning an intact version of IS*Aba1-bla*_ADC-195_ into the isolate 52971 could potentially restore ceftazidime resistance. As an intact version of *bla*_ADC-195_ was not available, a copy of its closest variant (*bla*_ADC-183_) carrying an upstream copy of IS*Aba1* was instead cloned into the plasmid pJN17/04 using primers presented in Table S3, as previously described (10), and this construct was electroporated into isolate 52971. Antimicrobial susceptibility testing results of selected transformants showed that they were able to grow directly next to a ceftazidime disk (no inhibition zone observed) on Mueller-Hinton agar plates (Fig. S2) and presented an increase in the ceftazidime MIC value from 1 mg/L to >64 mg/L.

Additional analysis of *bla*_ADC-195_ was performed to identify potential reasons for the insertion of IS*Aba1* within this gene. Interestingly, *bla*_ADC-195_ presented a synonymous G126A mutation (Fig. 1), increasing the AT content of the IS*Aba1* target site sequence, which might have contributed to its insertion (11). Nucleotide sequence alignment between *bla*_ADC-195_ and 327 publicly available *bla*_ADC_-like alleles (https://www.ncbi.nlm.nih.gov/pathogens/refgene/#blaADC, retrieved on 6th Nov 2024) revealed that such a polymorphism can only be found in 18% of *bla*_ADC_ gene variants (*n* = 59)*,* suggesting that the frequency of IS*Aba1* insertion in *bla*_ADC_-like might be low among *Acinetobacter* spp. isolates. In addition, it is worth noting that strains carrying ADC variants with the G126A mutation have been reported and associated with high ceftazidime MICs (8). However, the frequency of IS*Aba1* insertion disrupting other *bla*_ADC_ variants in *Acinetobacter* spp. and its effects on ceftazidime susceptibility remains to be determined.

In conclusion, this study described how IS*Aba1* was involved in reestablishing ceftazidime susceptibility in an extensively drug-resistant *A. baumannii* clinical isolate. Even though the results presented here were observed in a single isolate, the potential of IS to inactivate other chromosome-encoded resistance determinants should be further explored, as targeted insertions of this IS could potentially be used to recover the antimicrobial activity of ceftazidime and/or other drugs in multidrug-resistant *A. baumannii*.

## Data Availability

Raw reads of isolate 52971 were submitted to the Sequencing Read Archive of the National Center for Biotechnology Information (NCBI) under the BioSample number SAMN14929073. The sequence of the *bla*_ADC-195_ gene was deposited in NCBI beta-lactamases database under accession number MK840871.1.

## SUPPLEMENTAL MATERIAL

Supplemental Material aac.00707-25-s0001.pdf Tables S1 to S3; Fig. S1 and S2.

ASM does not own the copyrights to Supplemental Material that may be linked to, or accessed through, an article. The authors have granted ASM a non-exclusive, world-wide license to publish the Supplemental Material files. Please contact the corresponding author directly for reuse.

## References

[1] Adams MD, Goglin K, Molyneaux N, Hujer KM, Lavender H, Jamison JJ, MacDonald IJ, Martin KM, Russo T, Campagnari AA, Hujer AM, Bonomo RA, Gill SR. 2008. Comparative genome sequence analysis of multidrug-resistant Acinetobacter baumannii. J Bacteriol 190:8053–8064. doi:10.1128/JB.00834-08 18931120 PMC2593238

[2] Turton JF, Ward ME, Woodford N, Kaufmann ME, Pike R, Livermore DM, Pitt TL. 2006. The role of ISAba1 in expression of OXA carbapenemase genes in Acinetobacter baumannii. FEMS Microbiol Lett 258:72–77. doi:10.1111/j.1574-6968.2006.00195.x 16630258

[3] Potron A, Vuillemenot JB, Puja H, Triponney P, Bour M, Valot B, Amara M, Cavalié L, Bernard C, Parmeland L, Reibel F, Larrouy-Maumus G, Dortet L, Bonnin RA, Plésiat P. 2019. ISAba1-dependent overexpression of eptA in clinical strains of Acinetobacter baumannii resistant to colistin. J Antimicrob Chemother 74:2544–2550. doi:10.1093/jac/dkz241 31199431

[4] Mussi MA, Limansky AS, Viale AM. 2005. Acquisition of resistance to carbapenems in multidrug-resistant clinical strains of Acinetobacter baumannii: natural insertional inactivation of a gene encoding a member of a novel family of beta-barrel outer membrane proteins. Antimicrob Agents Chemother 49:1432–1440. doi:10.1128/AAC.49.4.1432-1440.2005 15793123 PMC1068641

[5] Gerson S, Nowak J, Zander E, Ertel J, Wen Y, Krut O, Seifert H, Higgins PG. 2018. Diversity of mutations in regulatory genes of resistance-nodulation-cell division efflux pumps in association with tigecycline resistance in Acinetobacter baumannii. J Antimicrob Chemother 73:1501–1508. doi:10.1093/jac/dky083 29554339

[6] Héritier C, Poirel L, Nordmann P. 2006. Cephalosporinase over-expression resulting from insertion of ISAba1 in Acinetobacter baumannii. Clin Microbiol Infect 12:123–130. doi:10.1111/j.1469-0691.2005.01320.x 16441449

[7] Bankevich A, Nurk S, Antipov D, Gurevich AA, Dvorkin M, Kulikov AS, Lesin VM, Nikolenko SI, Pham S, Prjibelski AD, Pyshkin AV, Sirotkin AV, Vyahhi N, Tesler G, Alekseyev MA, Pevzner PA. 2012. SPAdes: a new genome assembly algorithm and its applications to single-cell sequencing. J Comput Biol 19:455–477. doi:10.1089/cmb.2012.0021 22506599 PMC3342519

[8] Nodari CS, Cayô R, Streling AP, Lei F, Wille J, Almeida MS, de Paula AI, Pignatari ACC, Seifert H, Higgins PG, Gales AC. 2020. Genomic analysis of carbapenem-resistant Acinetobacter baumannii isolates belonging to major endemic clones in South America. Front Microbiol 11:584603. doi:10.3389/fmicb.2020.584603 33329450 PMC7734285

[9] Rodríguez-Martínez J-M, Poirel L, Nordmann P. 2010. Genetic and functional variability of AmpC-type β-lactamases from Acinetobacter baumannii. Antimicrob Agents Chemother 54:4930–4933. doi:10.1128/AAC.00427-10 20713667 PMC2976133

[10] Nowak J, Schneiders T, Seifert H, Higgins PG. 2016. The Asp20-to-Asn substitution in the response regulator AdeR leads to enhanced efflux activity of AdeB in Acinetobacter baumannii. Antimicrob Agents Chemother 60:1085–1090. doi:10.1128/AAC.02413-15 26643347 PMC4750716

[11] Mugnier PD, Poirel L, Nordmann P. 2009. Functional analysis of insertion sequence ISAba1, responsible for genomic plasticity of Acinetobacter baumannii. J Bacteriol 191:2414–2418. doi:10.1128/JB.01258-08 19136598 PMC2655512

