# Supplementary material for: ISAba1-mediated blaADC disruption restores third-generation cephalosporin susceptibility in a clinical Acinetobacter baumannii isolate
Source: Antimicrob Agents Chemother. 2025 Nov 18;69(12):e00707-25. doi: 10.1128/aac.00707-25 (PMC12691676; doi:10.1128/aac.00707-25)
Supplement: Supplemental Material — Tables S1 to S3; Fig. S1 and S2. [file aac.00707-25-s0001.pdf]

Table S1. Minimal inhibitory concentrations (MICs) and resistance determinants of *Acinetobacter baumannii* isolate 52971.

Antimicrobial Class	Antimicrobial Agent	MIC (mg/L)	Resistance determinants
**Cephalosporins**	Ceftazidime	1	-
**Carbapenems**	Imipenem	**128**	IS*Aba1*+*bla*$_{\mathrm{OXA\text{-}23}}$[a]
	Meropenem	**128**	
**Fluoroquinolones**	Ciprofloxacin	**>64**	*gyrA* (S83L, E87G), *parC* (S84L)
**Aminoglycosides**	Spectinomycin	ND	*aadA1*
	Gentamicin	**32**	*aac(3)-Ia*
	Amikacin	8	-
	Kanamycin	ND	*aph(3')-Ic*
**Sulphonamides**	Sulfamethoxazole	ND	*sul1*
**Tetracyclines**	Minocycline	2	-
**Glycylglycines**	Tigecycline	16	*adeS* (I100N, T156M)
**Polymyxins**	Polymyxin B	**32**	*pmrC* (L124S)
	Colistin	**>128**	

MIC values above BrCAST/EUCAST clinical breakpoints are highlighted in bold, when available. ND, not determined.

[a] In contrast to some class D beta-lactamases, the hydrolysis spectrum of OXA-23 does not include third-generation cephalosporins.

10.1128/aac.00707-25

Table S2. Whole-genome sequencing statistics for *A. baumannii* isolate 52971.

**Total length**	3,948,062 bp
**GC%**	39.09%
**Average coverage**	111x
**Number contigs (>500 bp)**	169
**Largest contig**	137,581 bp
**N50**	52,210 bp
**N75**	30,272 bp
**L50**	23
**L75**	48

Table S3. Primers used for the complementation assays performed in this study.

**Target**	**Primer name**	**Sequence (5′ - 3′)**	**Size (bp)**	**Experiment**
IS*Aba1*-ADC-183	P77	gccactcatcgcagtGCGCTGCATACGTCGATAAA	2155	InFusion cloning into pJN17/04
	P78	atgaattacaacagtCCGACTCAAGGCCATAATGC		
flanking *Sca*I	JE23	TTTGAGAAGCACACGGTCAC	452/2607	Detection of clone with insert
	JE24	TCATCCTGCCCTTATGTTCC		

Lowercase nucleotides indicate the tails for InFusion cloning.

10.1128/aac.00707-25

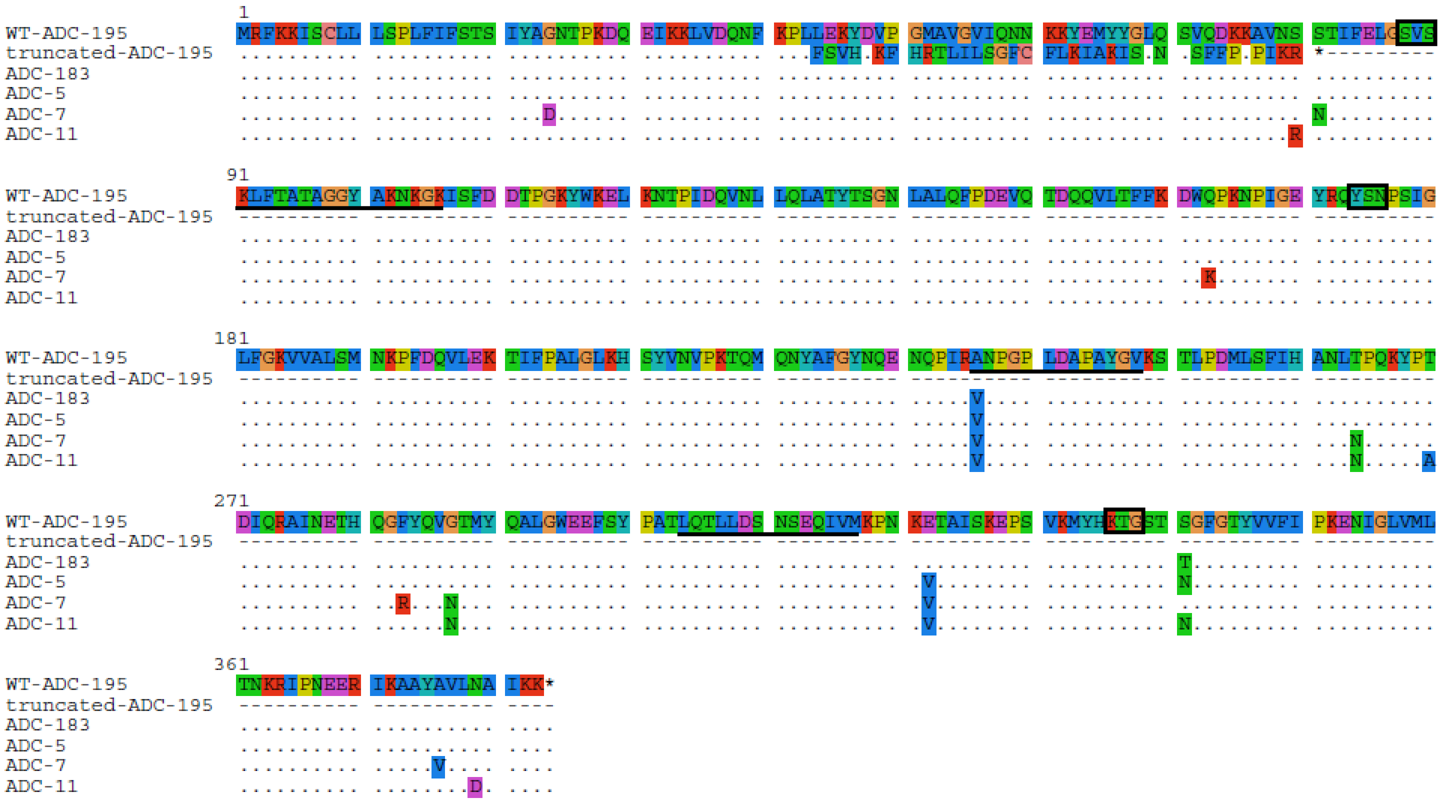

Fig. S1. Protein sequence alignment of WT and abnormal ADC-195 with ADC-183 and ADC-5, which presented the highest similarity (>99%) with ADC-195, as well as ADC-7 (reference for the ADC family) and ADC-11 (present in IC1 reference strain AYE). Conserved amino acid identities are indicated by dots, motifs associated with beta-lactamase activity are highlighted in the boxes, and H2-, H10-helices and the Ω-loop are underlined.

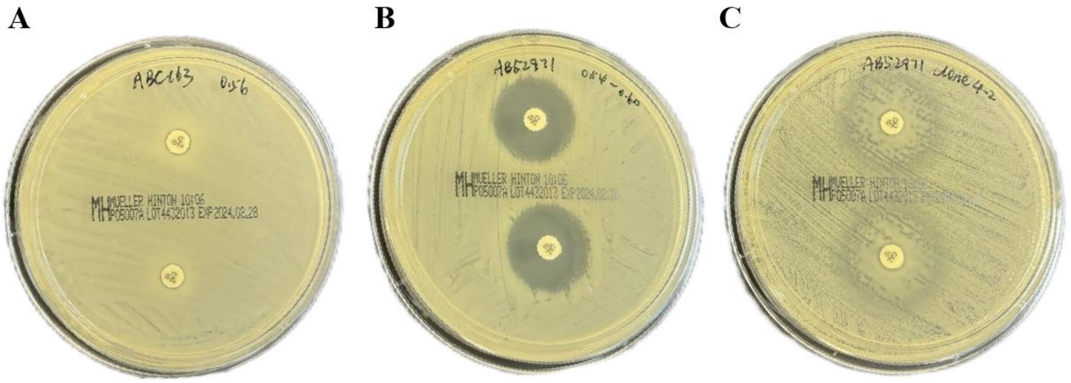

Fig. S2. Ceftazidime susceptibility testing results (ceftazidime disks in duplicate on each plate). **(A)** Isolate ABC163 containing IS*Aba1*-*bla*$_{\text{ADC-183}}$ showing no inhibition zone for ceftazidime; (B) Isolate 52971 containing *bla*$_{\text{ADC-195}}$ disrupted by IS*Aba1* showing an inhibition zone of 22 mm for ceftazidime; **(C)** Isolate 52971 containing the construct pJN17/04::IS*Aba1*-*bla*$_{\text{ADC-183}}$ showing growth in the inner zone of inhibition of ceftazidime disk.

10.1128/aac.00707-25
